# Sympathetic Ophthalmia Following an Evisceration Surgery—A Case Report

**DOI:** 10.1002/ccr3.9626

**Published:** 2024-11-27

**Authors:** Rahmah Javed, Saba Al‐Khairy, Hamna Khan, Hamna Raheel, Abdul Moeed, Salim Surani

**Affiliations:** ^1^ Dow Medical College Dow University of Health Sciences Karachi Pakistan; ^2^ Texas A&M University System Health Science Center College Station Texas USA

**Keywords:** case report, evisceration, panuveitis, rare, sympathetic ophthalmia

## Abstract

Sympathetic ophthalmia (SO) is a rare, bilateral, granulomatous, panuveitis following perforating trauma or surgical intervention in one eye. Here, and to the best of knowledge, we report the first case of SO following an evisceration surgery in Pakistan. A 32‐year‐old, Pakistani, female presented with pain and decreased visual acuity in her right eye, at Civil Hospital, Karachi, 1 week after an evisceration surgery was performed on her left eye, following corneal perforation due to unresolved keratitis. Diagnosis of SO was made on the basis of clinical presentation and confirmed by slit lamp examination which revealed mutton fat keratic precipitates, posterior synechiae, and optic disc swelling. She was administered high‐dose corticosteroids to which she did not respond. She was then prescribed methotrexate which resolved the inflammation and improved visual acuity. Initiating prompt treatment for the sympathizing eye led to effective management of the patient and prevented complete blindness.

**Abbreviations**
HLA: human leukocyte antigen
OPD: outpatient department
SO: sympathetic ophthalmia
VKHD: Vogt–Koyanagi–Harada disease
YAG: yttrium aluminum garnet

**Summary**

- Early detection and management of sympathetic ophthalmia (SO), especially following intraocular procedures or trauma, is imperative in preventing potentially fatal outcomes in most patients.
- This patients' presentation demonstrates the importance of counseling patients regarding SO as the possible adverse effect of their surgery and the need for more evidence‐based management, which currently relies heavily on steroids and immunosuppressants.

## 1 Introduction

Sympathetic ophthalmia (SO) is inflammation that originates in an injured or operated eye and extends to the adjacent eye. It is usually referred to as bilateral, granulomatous panuveitis. The opposite eye is known as the sympathizing eye, whereas the traumatized eye is the instigating eye [1]. It is commonly associated with, but not limited to [2], penetrating or perforating injury to an eye. Despite its association with penetrating trauma, it has also been found in patients following intraocular surgeries such as cataract extraction, glaucoma filtration surgery, scleral buckling, cyclo‐destructive surgeries, squint surgeries, pars plana vitrectomy, and trans conjunctival suture less vitrectomy, with the latter two having a more significant association [3, 4, 5, 6]. Several non‐penetrating eye procedures have also been reported to be associated with SO which include neodymium: yttrium aluminum garnet (YAG) laser cyclo destruction, laser cyclo cryotherapy, proton beam irradiation, and Ruthenium plaque brachytherapy [2].

In a recent meta‐analysis conducted by Bondok et al. [5], the overall incidence of SO following intraocular procedures was 0.061% (95% CI: 0.033%–0.111%) yielding a statistically significant result. Another study conducted by Patterson et al. reported that there was no risk of developing SO postevisceration or enucleation. Although SO has been reported following evisceration procedures [7, 8, 9], the amount of information available is still scarce regarding detection and management. Therefore, to help bridge the gap in literature and the confounding data available we present a case of SO following an evisceration surgery. This, to the best of our knowledge, is the first case of SO to be reported in Pakistan.

This case report has been reported in accordance with the CARE criteria [10].

## 2 Case Presentation

**FIGURE 1:**
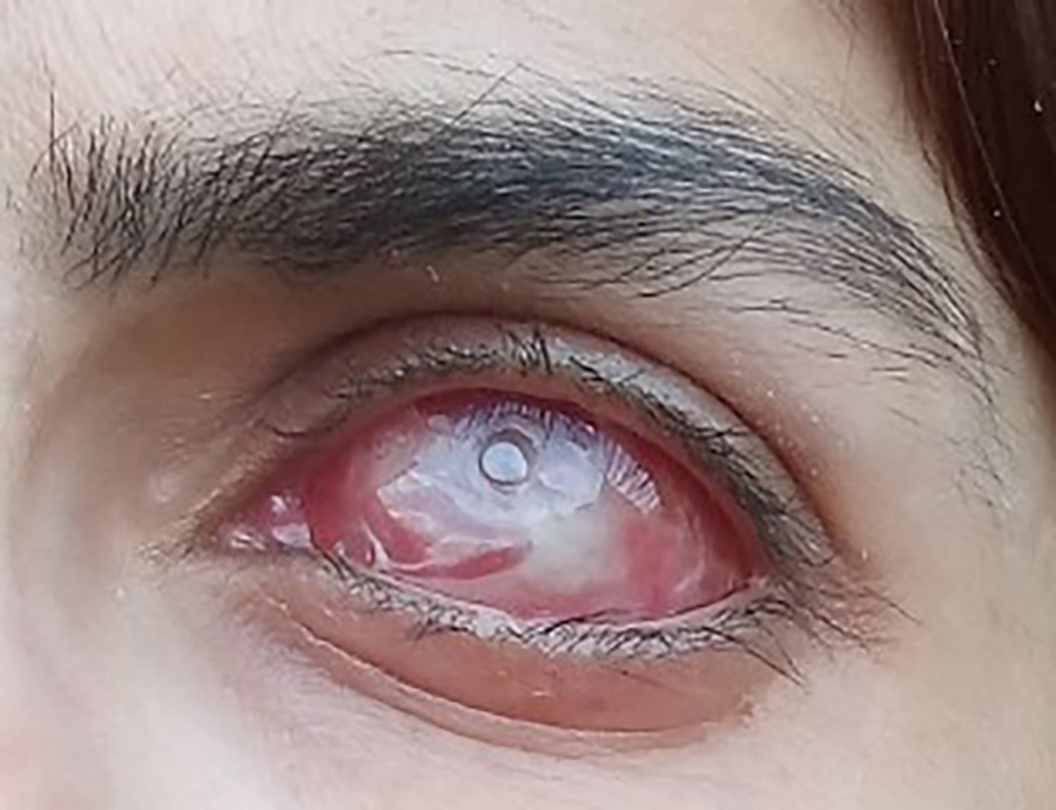
Patient's left eye postevisceration.

A 32‐year‐old, Pakistani, female presented to the eye outpatient department (OPD) of Civil Hospital in Karachi, Pakistan, with left eye pain and redness. She had a history of having worn contact lenses for a few hours in December. No relevant past medical history, family history, drug history, allergies, or addictions were noted. Slit lamp examination revealed keratitis in her left eye with an abscess measuring 3 × 4 mm and a 2 mm hypopyon. She was treated for keratitis with Moxifloxacin 0.5% (every 2 h) and Brolene 0.1% (4 times a day) eye drops. Her condition did not improve, and she ended up with corneal perforation. Vision dropped to no light perception and the eye continued to be painful. Subsequently, an evisceration surgery was performed in January. Her eviscerated eye was cosmetically rehabilitated with a prosthetic eye. The pain resolved postoperatively and the patient was discharged (Figure 1). One week postoperatively, she presented with pain in her right eye, photophobia, and blurred vision. Upon examination, her visual acuity had reduced to 6/60. Further examination revealed mutton fat keratic precipitates +2 cells in the anterior chamber and posterior synechiae over the pupillary membrane. Posterior segment examination revealed vitritis +3 cells along with optic disc swelling. The retina was normal.

## 3 Differential Diagnoses

Given that TB is widespread in Pakistan, tuberculosis uveitis, Vogt–Koyanagi–Harada disease (VKHD), and sarcoidosis were among the differential diagnoses that were suspected at the time of her presentation. Given the absence of any indications of these conditions, the patient's lack of immunocompromised history, and the fact that she had already undergone surgery on the opposite eye, SO was considered the most likely diagnosis.

## 4 Management

**FIGURE 2:**
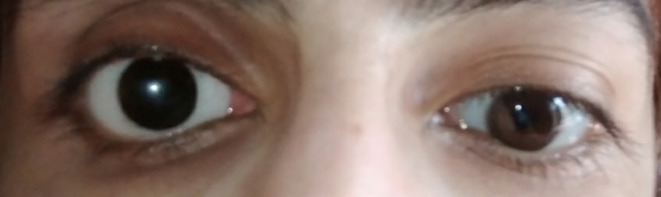
Patient's current status.

The patient was prescribed high‐dose, topical (prednisone 1% drop every 2 h), and oral (Deltacortil 5 mg, 5 tablets in the morning and evening) corticosteroids 1 mg/kg/day and told to follow up 1 week later. In addition, she was administered three daily doses of 1% cyclopentolate HCL and 1 mg/mL nepafenac eye drops. Upon following up, her symptoms had not improved. She was then prescribed methotrexate, one dose of 7.5 mg per week, for 2 months, which resolved the inflammation and her symptoms. Her visual acuity improved from 6/60 to 6/9 and no signs of inflammation were present posttreatment. The patient did not have any adverse reactions to the medication and showed significant improvement in symptoms following the administration of methotrexate (Figure 2).

## 5 Discussion

SO is a rare inflammatory condition commonly associated with penetrating trauma and intraocular surgeries. The precise incidence and prevalence of SO are difficult to determine because of its rarity. In the meta‐analysis performed by Bonnie et al., the overall incidence following open globe injury was 0.19% [11]. According to the study conducted by Marak et al. [12], the incidence of SO following intraocular surgery was 0.1% and 0.2%–0.5% following penetrating injury. Although SO has been associated with multiple types of intraocular procedures, it has been infrequently reported postevisceration surgery [7].

The time interval between the precipitating event of the inciting eye and the onset of symptoms in the sympathizing eye varies considerably, ranging from only a few days to decades at a stretch. According to previous reports, 17% of the cases manifested within 1 month, 50% within 3 months, 65% within 6 months, and 90% within the first year [1].

The common symptoms, which develop progressively, include near vision disturbances, pain, photophobia, floaters, and redness [3]. Clinical features often seen in cases of SO under slit lamp examination are conjunctival congestion, mutton fat keratic precipitates on the corneal endothelium, and anterior chamber reaction. With fundus examination, vitritis, serous retinal detachment, choroiditis, papillitis, optic disc edema, retinal vasculitis, and Dalen–Fuchs nodules (clusters of epithelioid cells containing pigment lying between the retinal pigment epithelium and Bruch's membrane) are often seen. Most of the above‐mentioned features, however, are not limited to SO, and the differential diagnoses may include VKHD, tuberculosis, sarcoidosis, syphilis, and lymphoma [13].

Possible etiologies include genetic predisposition to developing SO with a particular focus on human leukocyte antigens (HLA) and the formation of anti‐uveal antibodies. It has particularly been associated with HLA‐A11 antigen along with HLA‐DRB1*04, DQA1*03, and DQB1*04. Lymphatics have also been suspected to play a role in the manifestation of SO. Following trauma to the eye, intraocular antigens can travel to the regional lymph nodes leading to the development of a cell‐mediated immune response, mitigated by CD4+ helper T cells, to the uvea and the retinal photoreceptor layer [14].

Following her diagnosis, the patient was prescribed high‐dose corticosteroids, which is the first‐line treatment indicated for SO. However, upon following up a week later her symptoms had not improved. Since the patient was already one‐eyed, methotrexate was then prescribed as second‐line therapy to preserve her vision and prevent blindness. Steroids and immunosuppressants have long been the mainstay of treatment for SO, although these medications do have promising outcomes, long‐term use can cause significant adverse effects and immunosuppression. The rarity of the condition, along with limited research conducted regarding the pathophysiology, has led to limited clinical trials and therefore scarcity of data available regarding the best therapy specifically targeted toward SO [15, 16, 17].

## 6 Conclusion

SO, although rare, continues to be a significant issue since a definitive cause is yet to be identified and, in most cases, the condition can be disabling. Patients undergoing intraocular procedures should regularly be counseled regarding the condition. A high index of suspicion is required by the physician to initiate prompt treatment and prevent potential blindness in the patient.

## Author Contributions

**Rahmah Javed:** conceptualization, writing – original draft. **Saba Al‐Khairy:** conceptualization, writing – original draft. **Hamna Khan:** writing – original draft. **Hamna Raheel:** writing – original draft. **Abdul Moeed:** writing – review and editing. **Salim Surani:** supervision, writing – review and editing.

## Ethics Statement

The authors have nothing to report.

## Consent

Written informed consent was obtained from the patient to publish this report in accordance with the journal's patient consent policy.

## Conflicts of Interest

The authors declare no conflicts of interest.

## Data Availability

The datasets used and/or analyzed during the current study are available from the corresponding author upon reasonable request.
